# Pregnancy cohorts and biobanking in sub-Saharan Africa: a systematic review

**DOI:** 10.1136/bmjgh-2020-003716

**Published:** 2020-11-26

**Authors:** Jeffrey N Bone, Kelly Pickerill, Mai-Lei Woo Kinshella, Marianne Vidler, Rachel Craik, Lucilla Poston, William Stones, Esperanca Sevene, Marleen Temmerman, Angela Koech Etyang, Anna Roca, Donna Russell, Rachel M Tribe, Peter von Dadelszen, Laura A Magee, The PRECISE Network, Umberto D’Alessandro

**Affiliations:** 1 Department of Obstetrics and Gynaecology, The University of British Columbia, Faculty of Medicine, Vancouver, British Columbia, Canada; 2 Department of Women and Children’s Health, King's College London, School of Life Course Sciences, Faculty of Life Sciences and Medicine, London, UK; 3 Centre for Reproductive Health, University of Malawi College of Medicine, Blantyre, Malawi; 4 Centro de Investigação em Saúde de Manhiça, Manhiça, Maputo, Mozambique; 5 Department of Physiologic Sciences, Faculty of Medicine, Universidade Eduardo Mondlane, Maputo, Mozambique; 6 Department of Obstetrics and Gynaecology, Centre of Excellence in Women and Child Health, Aga Khan University, Nairobi, Kenya; 7 Medical Research Council Unit, The Gambia at the London School of Hygiene and Tropical Medicine, Fajara, The Gambia; 8 Donna Russell Consulting, Seattle, Washington, USA

**Keywords:** systematic review, cohort study, obstetrics, maternal health

## Abstract

**Background:**

Technological advances and high throughput biological assays can facilitate discovery science in biobanks from population cohorts, including pregnant women. Biological pathways associated with health outcomes differ depending on geography, and high-income country data may not generalise to low-resource settings. We conducted a systematic review to identify prospective pregnancy cohorts in sub-Saharan Africa (SSA) that include biobanked samples with potential to enhance discovery science opportunity.

**Methods:**

Inclusion criteria were prospective data collection during pregnancy, with associated biobanking in SSA. Data sources included: scientific databases (with comprehensive search terms), grey literature, hand searching applicable reference lists and expert input. Results were screened in a three-stage process based on title, abstract and full text by two independent reviewers. The review is registered on PROSPERO (CRD42019147483).

**Results:**

Fourteen SSA studies met the inclusion criteria from database searches (n=8), reference list searches (n=2) and expert input (n=4). Three studies have ongoing data collection. The most represented countries were South Africa and Mozambique (Southern Africa) (n=3), Benin (Western Africa) (n=4) and Tanzania (Eastern Africa) (n=4); including an estimated 31 763 women. Samples commonly collected were blood, cord blood and placenta. Seven studies collected neonatal samples. Common clinical outcomes included maternal and perinatal mortality, malaria and preterm birth.

**Conclusions:**

Increasingly numerous pregnancy cohorts in SSA that include biobanking are generating a uniquely valuable resource for collaborative discovery science, and improved understanding of the high regional risks of maternal, fetal and neonatal morbidity and mortality. Future studies should align protocols and consider their added value and distinct contributions.

**Key questions**

**What is already known?**

- Availability of large-scale biorepositories and advances in ‘omics technology have opened up the possibility of discovery science to better understand adverse maternal and neonatal outcomes.
- Sub-Saharan Africa has the highest burden of such outcomes globally, and the creation of biobanks is a rapidly increasing field of research to support interventions to reduce these outcomes.

**What are the new findings?**

- This is the first systematic review to specifically focus and characterise all sub-Saharan African pregnancy cohorts that include biological samples stored for future research.
- The majority of these cohorts have been established within the last decade and together provide a rich set of biological samples such as blood, cord blood and placenta, to aid global health researchers in better understanding disease pathways in African women.
- Most of these cohorts include women who are attending facilities, and do not provide long-term maternal-infant follow-up.

**What do the new findings imply?**

- Given the time, resources and efforts required—by both researchers and participants—to contribute, collect and store biological samples, future projects in sub-Saharan Africa planning to establish biobanks can offer value through complementing existing cohorts.
- Further, this field would benefit from continued standardisation of protocols for biobanking to enable data sharing and collaboration, and careful consideration of global health research priorities.

## Introduction

Recently, the proliferation of fast throughput biological assays and ‘omics technologies (genomics, epigenomics, metabolomics, lipidomics and proteomics), as applied to large biorepositories, has enabled discovery science at scale.^1^ Studies that include biorepositories facilitate the study of relationships between clinical and environmental data, outcomes and multilayered biological variables—at both individual and population levels.^2^ This approach has the capacity to provide a holistic picture of health and shed light on the biological mechanisms underlying both health and disease.^3^

This approach is essential in maternal and perinatal research as the relationships between adverse pregnancy/birth outcomes, environmental exposures (eg, climate, nutrition, infection) and genome and phenotypic characteristics are only beginning to be understood.^4^ Researchers in higher resourced settings such as the United Kingdom, the Netherlands and Scandinavia have established large pregnancy cohorts and biobanks to help address these questions.^5–8^ However, there are challenges in applying the results from these and other high-income country studies, to low and middle-income countries, where the biological mechanisms, risk factors, environmental exposures and clinical outcomes may differ.^9 10^

Sub-Saharan Africa (SSA) has the highest burden of maternal, perinatal and child deaths globally,^11 12^ yet little is known about the complex interactions between context-specific exposures and the biological mechanisms that inevitably underlie this excess in deaths.^9 10^ Better understanding of these processes would guide prevention, screening and treatment measures, and has the potential to inform resource allocation and distribution, as well as health policy at regional and country levels.

To determine the extent of biobanking and discovery science in SSA, current gaps and areas the global health community should prioritise, we conducted a systematic review of pregnancy cohorts that include biological samples stored in biorepositories for future research use. In light of the recent shift in focus of global health research to centre around the COVID-19 pandemic, such studies are particularly pertinent as they will provide valuable resources to generate important knowledge.

## Methods

This review has been developed in accordance with the Preferred Reporting Items for Systematic Reviews and Meta-Analyses (PRISMA) checklist^13^ and is registered on PROSPERO (CRD42019147483). The PRISMA checklist is included as Online Supplemental File 1.

10.1136/bmjgh-2020-003716.supp2 Supplementary data

### Objective

Our objective was to systematically review pregnancy cohort studies that include biobanking in SSA. For our purposes outlined here, biobanking was broadly defined to include any biological sample collected and stored for future use—excluding samples collected solely for point-of-care testing. Additionally, the review aimed to characterise the study designs of the cohorts, their populations, exposures and outcomes reported, as well as to examine the completeness of participant follow-up. By determining the number and depth of existing biobanks, this review provides a resource for addressing current knowledge gaps. Identifying these gaps is pivotal in order to generate new evidence for improving maternal and newborn health in the region—and for framing recommendations for future cohort study-based research and biobanks.

### Study inclusion and exclusion

All prospective studies of pregnant women in SSA conducted between 1 January 2000 and 30 September 2019 that collected clinical, and if relevant, sociological data, and have biobanked samples for future use were eligible for inclusion. While we expected most cohorts to be recent due to technological development in metabolomics and research inclusive of deep phenotyping, we extended our search back to 2000 to ensure no earlier cohorts were missed. Studies were assessed by reviewing study sampling methods, laboratory protocols or standard operating procedures (SOPs) for explicit indication that samples were collected and stored for future use, or for subsequent immediate analysis. In cases (two) where this was unclear, we approached the study authors for clarification on sample storage indication and purpose of sample use. Both randomised and observational studies were included. Randomised trials were included as often the data sets/samples would be available as a ‘cohort’ for secondary analyses. Exclusion criteria consisted of cohorts collecting biological samples exclusively from non-pregnant women, studies outside SSA, studies collecting samples for point-of-care assays only and studies conducted prior to the year 2000. In the case of multiple articles referencing the same study/cohort, only one report with the most comprehensive information was included, but data from multiple publications may have been collated (ie, from both the protocol and subsequent publications).

### Search strategy

The primary search was conducted in MEDLINE, Embase, Web of Science, Africa Index Medicus and CAB Direct. A combination of Medical Subject Headings (MeSH) terms related to geographical location (SSA), pregnancy and pregnancy-related adverse outcomes and biological samples was used. Search terms are included in Online Supplemental Table S2. All searches were conducted from July through September 2019, with the search date cut-off excluding studies published after 30 September 2019.

10.1136/bmjgh-2020-003716.supp1 Supplementary data

Results were screened in a three-stage process based on title, abstract and then full-text review. At each stage, all titles, abstracts and full papers were reviewed by two independent reviewers (JB, KP), and in the case of disagreement, a third reviewer (MLWK) assisted to break the tie. Duplicates were excluded using R statistical software V.3.5.3.^14^ Additional articles were identified through hand searching the references of included papers, and input from experts in the field.

### Data extraction

Data were extracted independently by the two reviewers and recorded using an Excel spreadsheet. In the case of disagreements these were resolved via discussion, and if necessary, the intervention of the third reviewer (MLWK). The data extracted from each included study comprised year(s) of publication and active data collection, geography and study design, samples collected, and maternal and perinatal mortality and morbidities.

### Data analysis

Data regarding the volume collected, storage method and timing of each biological sample were summarised. For each biological sample, the proportion of studies collecting this sample was reported. Storage and gestational timing of collection information was similarly summarised. If any biological sample was collected (with collection data) in at least two studies, then the rates of ascertainment (total participants with sample/total participants in study) were combined with mixed effects logistic regression, treating the study as a random effect. In these cases, the overall rate and 95% confidence intervals (CIs) were reported, together with I² statistics, for heterogeneity. Clinical outcomes with rates reported in at least two studies were also combined. All statistical analyses were conducted using R version V.3.5.3.^14^

### Quality and risk of bias assessment

Each included study was assessed for quality using the Newcastle-Ottawa Scale for cohort studies (http://www.ohri.ca/programs/clinical_epidemiology/oxford.asp) according to selection of the study group (score 0–4), comparability of the groups (score 0–2) and ascertainment of outcomes (score 0–3). Complete details are given in Online Supplemental Table S3. A PRISMA checklist^13^ for this review is included (Online Supplemental File 1).

### Patient and public involvement

Patient/public involvement was unfortunately not feasible in this review.

## Results

**Figure 1:**
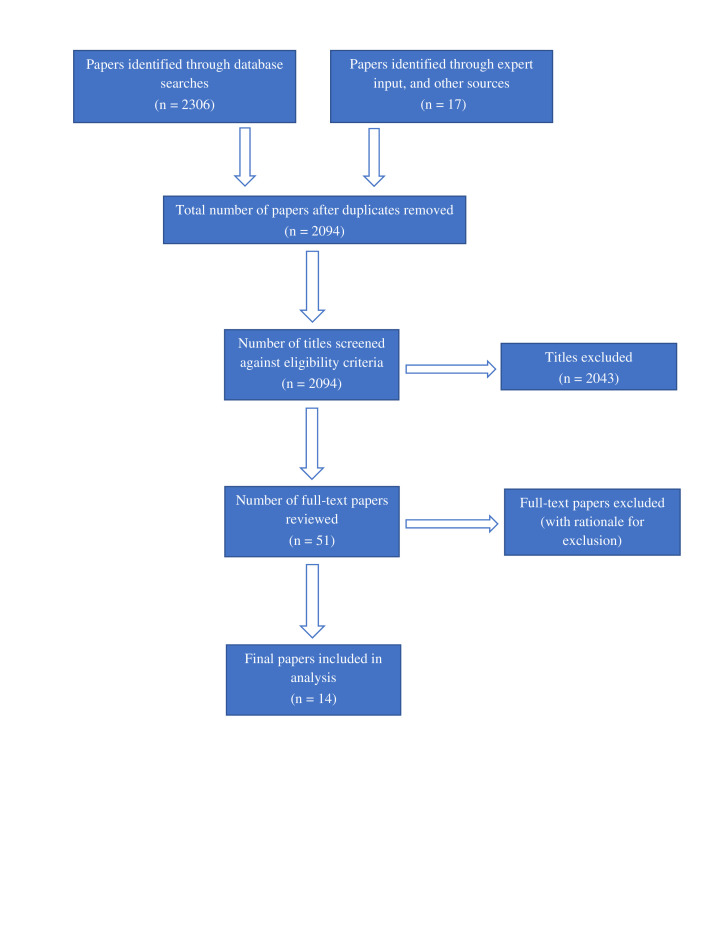
Study eligibility and inclusion flow chart.

In total, 14 cohorts were identified that met the inclusion criteria.^9 10 15–27^ Eight of these were identified from the search terms and strategy outlined above, two from hand searching references of identified articles and two via expert input. For one of the four studies identified through expert input,^17^ the protocol is pending publication. See figure 1 for details.

Studies were spread across eight countries, with Tanzania and Benin each being the most common location (with four studies in each country). Five studies were multicountry (within SSA), four of these^9 15 19 22^ being the three largest included (n=2032, 4732, 4749 and 11 800, respectively). There were also two studies^10 27^ that included both sites inside and outside SSA, mainly in South Asia. Nine had completed data collection and published details, with a median sample size of 953 (IQR=472–2707); the smallest had 250 participants.^24^ The Bill & Melinda Gates Foundation was the most common funder, with three studies acknowledging this organisation as the sole or partial funder. Otherwise, funding sources were predominately from either European countries or the European Union, with the smallest cohort^24^ funded by the South African Medical Research Council. All but one^16^ protocol/study were published within the past 10 years, with five studies published in 2019, and one in 2020. Cohorts were broadly similar in terms of their inclusion criteria and target populations. Full details of each included publication are shown in table 1.

**Table 1:** Cohorts and study characteristics of pregnancy cohorts identified from review

Study title	Country	Years of recruitment	Number of subjects*	Study design	Antepartum sample data collection	Delivery sample data collection	Postpartum sample data collection	Neonatal/paediatric samples collected	Primary clinical outcome	Data collection complete
IPTp +: Intermittent Preventive Treatment in Pregnant Women in the Context of Insecticide Treated Nets^21^	Mozambique	2003-2006	1030	Randomised control trial	Y	Y	Y	Y	Low birth weight	Y
STOPPAM: Strategies to Prevent Pregnancy Associated Malaria^24^	Tanzania, Benin	2008-2010	2037	Prospective cohort	Y	Y	N	N	Malaria	Y
Seychelles Child Development Study^23^ †	Seychelles	2008-2011	1535	Prospective cohort	Y	N	Y	Y	Child neurodevelopment at 20 months	Y
MiPADD trial: Malaria in Pregnancy Preventive Alternative Drugs^20^	Benin, Gabon, Mozambique and Tanzania	2009-2013	4739	Randomised control trial	Y	Y	Y	Y	Low birth weight	Y
ENID trial: early nutrition and immune development^18^	The Gambia	2010-2015	875	Randomised control trial	Y	Y	Y	Y	Infant thymic size	Y
COSMIC: Community-based Malaria Screening and Treatment for Pregnant Women Receiving Standard Intermittent Preventive Treatment With Sulfadoxine-Pyrimethamine^27^	Burkina Faso, The Gambia, and Benin	2013-2015	4731	Cluster randomised control trial	Y	Y	N	N	Placental malaria	Y
FOETAL for NCD - FOetal Exposure and Epidemiological Transitions^17^	Tanzania	2014-2015	538	Prospective cohort	Y	Y	Y	N	Anaemia	Y
Effect of malaria in early pregnancy on fetal growth in Benin (RECIPAL preconceptional cohort)^15^	Benin	2014-2016	982	Prospective cohort	Y	Y	N	N	Foetal growth	Y
AMANHI bio-banking^10^	Tanzania	2014-2016	1000	Prospective cohort	Y	Y	Y	Y	Pre-eclampsia, growth restriction, preterm birth, stillbirth	Y
INTERBIO 21st Newborn^19^	Kenya, South Africa	2012-2018	NA	Case-control	N	Y	N	N	Preterm birth, small for gestational age	Y
Nutrition during pregnancy and early development (NuPED)^16^	South Africa	2016-2019	250	Prospective cohort	Y	Y	Y	N	Nutritional status	Y
The Zambian Preterm Birth Prevention Study (ZAPPS)^26^	Zambia	2015 - present	1450	Prospective cohort	Y	Y	N	Y	Preterm birth	N
The PRECISE (PREgnancy Care Integrating translational Science, Everywhere)^9^	The Gambia, Kenya, Mozambique	2019-present	11800	Prospective cohort	Y	Y	Y	Y	Placental disorders of pregnancy (pregnancy hypertension, foetal growth restriction, and stillbirth)	N
HeLTI: Healthy Life Trajectories Initiative^22^ ‡	South Africa	2020-present	1500	Randomised control trial	Y	Y	Y	Y	Adiposity	N

*In cases where study data collection is not complete, this represents published sample size target.

†Here we used the paper “Maternal immune markers during pregnancy and child neurodevelopmental outcomes at age 20 months in the Seychelles Child Development Study”, as it is the most recent relevant publication from this large ongoing Seychelles 2 Study.

‡Pendingpublication.

All protocols, except for the Functional Classification of Abnormal Fetal and Neonatal Growth Phenotypes, Newborn Case Control Study (INTERBIO-21st), had multiple sampling points, and all but the Seychelles Child Development Study collected samples at delivery. Nine included postpartum sampling.^9 10 15–18 24–26^ The samples most commonly collected were maternal blood (14 studies) and placenta (11 studies). Where reported, ascertainment rates of sample collection from participants were high; for example, the pooled rate of women providing at least one blood sample was 92.0% (95% CI 83.1% to 96.4%). Hair, vaginal swabs, tissue and breast milk were less frequently collected (≤2 studies in all cases). Eight studies reported collection of neonatal samples,^9 10 15–18 21 26^ and of these, cord blood and neonatal blood were most common. Table 2 provides details of sample collection.

**Table 2:** Biobanking information (sample type proportions, and timing)

Sample	Number of studies collecting	Collected antepartum	Collected at delivery	Collected postpartum	Reporting number of subjects with sample*	Proportion of subjects with at least one sample collected (weighted)†	I^2^
Blood	14 (100%)^9 10 15–19 22–24 26 27^	13 (92.9%)	9 (64.3%)	8 (57.1%)	7 (50.0%)	92.0% (83.1%, 96.4%)	99.6%
Vaginal swab	2 (14.2%)^9 26^	2 (14.2%)	2 (14.2%)	0 (0.0%)	0 (0.0%)	–	–
Hair	1 (7.1%)^23^	1 (7.1%)	0 (0.0%)	0 (0.0%)	0 (0.0%)	–	–
Cord blood	11 (78.6%)^9 10 15 16 18–22 24^	–	11 (78.60%)	NA	4 (36.4%)	70.3% (47.5%, 86.0%)	99.6%
Urine	7 (50.0%)^9 10 15 16 18 22 26^	7 (50.0%)	6 (42.9%)	6 (42.9%)	0 (0.0%)	–	–
Stool	3 (21.4%)^10 15 19^	2 (14.2%)	3 (21.4%)	1 (7.1%)	0 (0.0%)	–	–
Placenta	11 (78.6%)^9 10 15 17–21 24 26 27^	–	11 (78.6%)	NA	5 (45.4%)	68.0% (49.0%, 82.4%)	99.7%
Tissue	2 (16.6%)^10 26^	2 (14.2%)	2 (14.2%)	1 (7.1%)	0 (0.0%)	–	–
Breast milk	3 (25.0%)^16 18 22^	–	–	3 (25.0%)	0 (0.0%)	–	–
Neonatal urine	0 (0.0%)	–	–	0 (0.0%)	0 (0.0%)	–	–
Neonatal stool	2 (16.6%)^9 10^	–	–	2 (16.6%)	0 (0.0%)	–	–
Neonatal blood	6 (42.9%)^9 18 20–22 26^	–	–	6 (42.9%)	1 (16.6%)	–	–

*Denominatoris number on studies collecting corresponding sample.

†Based onrandom effects logistic regression model.

The gestational time of sampling varied across cohorts, and also differed by sample type. As illustrated in table 3, blood sample collection occurred primarily in the antenatal period, with 9, 10 and 11 studies collecting samples in the first, second and third trimesters, respectively. Nine studies collected blood at delivery and six collected blood post partum (see table 3); many also collected cord blood and/or placenta samples at delivery (see table 2).

**Table 3:** Timing of maternal blood samples across studies

Study	Pre-pregnancy	First trimester	Second trimester	Third trimester	Delivery	48 hours postpartum	6 weeks postpartum	6 months postpartum	12 months postpartum	60 months postpartum
IPTp +^21^		X	X	X	X		X			
STOPPAM^24^		X	X	X	X					
Seychelles Child Development Study^23^			X							
MiPADD^20^		X	X	X	X		X			
ENID^18^			X	X						
COSMIC^27^			X	X	X					
FOETAL^17^		X	X	X						
RECIPAL^15^	X	X		X						
AMANHI^10^		X	X	X		X	X			
INTERBIO-21st^19^					X					
NuPED^16^		X	X	X	X			X	X	
ZAPPS^26^		X	X	X	X					
PRECISE^9^		X	X	X	X	X	X			
HeLTI^22^	X	X	X		X					X

X indicates data is collected at that time point.

Most studies recorded all the principal clinical outcomes outlined for data extraction, with maternal mortality, anaemia, stillbirth and neonatal death being the most common. Despite this, as many are either ongoing, or have yet to publish primary results, few have reported outcome data on those outcomes we described a priori. Where data were available, we pooled outcome rates and found a maternal mortality ratio of 120 (95% CI 80 to 280; 24 women, 8 studies, I^2^=14.6%) per 100 000 births, and stillbirth and neonatal death rates of 20 (95% CI 15 to 28; 297 fetuses, 7 studies, I^2^=84.6%) and 11 (95% CI 6 to 20; 154 infants, 5 studies, I^2^=89.1%) per 1000 total and live births, respectively. Pregnancy hypertension, maternal anaemia and malaria had rates of 3.4% (95% CI 2.6% to 4.4%, 59 women, 2 studies, I^2^=NA), 37.4% (95% CI 22.0% to 55.9%, 4868 women, 8 studies, I^2^=99.1%) and 5.2% (95% CI 0.90% to 24.4%, 440 women, 4 studies, I^2^=99.5%), respectively. Full details of clinical outcomes are provided in table 4. Additional outcomes collected in at least two studies—but not presented here—were HIV and diabetes (two studies each), and low birth weight (four studies). Only three studies had long-term (greater than one year) maternal or paediatric outcomes.

**Table 4:** Social and clinical determinants of health and clinical outcomes studied

Variables and outcome	Number of studies collecting	Studies collecting*	Number of studies reported†	Studies reporting	Weighted proportion of outcome (95% CI)‡	*I^2^*
Social determinants of health						
Health system	5 (35.7%)	COSMIC,^27^ FOETAL,^17^ RECIPAL,^15^ AMANHI,^10^ PRECISE^9^	3 (60.0%)	COSMIC,^27^ FOETAL,^17^ RECIPAL^15^	–	–
Women’s status in society	10 (71.4%)	IPTp +,^21^ Seychelles Child Development Study^23^ COSMIC,^27^ FOETAL,^17^ RECIPAL,^15^ AMANHI,^10^ NuPED,^16^ ZAPPS,^26^ HeLTI,^22^ PRECISE^9^	5 (50.0%)	Seychelles Child Development Study,^23^ COSMIC,^27^ FOETAL^17^ RECIPAL,^15^ ZAPPS^26^	–	–
Health geography (including air pollution and WASH)	7 (50.0%)	IPTp +,^21^ COSMIC,^27^ FOETAL,^17^ AMANHI,^10^ ZAPPS,^26^ HeLTI,^22^ PRECISE^9^	3 (42.8%)	COSMIC,^27^ FOETAL,^17^ ZAPPS^26^	–	–
Nutrition	7 (50.0%)	ENID,^18^ RECIPAL,^15^ AMANHI,^10^ INTERBIO-21st,^19^ NuPED,^16^ HeLTI,^22^ PRECISE^9^	–	–	–	–
Clinical determinants of health						
Infectious disease burden	12 (85.7%)	IPTp +,^21^ STOPPAM,^24^ ENID,^18^ COSMIC,^27^ MiPADD,^20^ FOETAL^17^ RECIPAL,^15^ AMANHI,^10^ INTERBIO-21st,^19^ NuPED,^16^ ZAPPS,^26^ PRECISE^9^	7 (58.3%)	IPTp +,^21^ STOPPAM,^24^ COSMIC,^27^ MiPADD,^20^ FOETAL,^17^ RECIPAL,^15^ ZAPPS^26^	–	–
Non-communicable disease burden	10 (71.4%)	COSMIC,^27^ ENID,^18^ FOETAL,^17^ RECIPAL,^15^ AMANHI,^10^ INTERBIO-21st,^19^ NuPED, ZAPPS,^26^ HeLTI,^22^ PRECISE^9^	4 (40.0%)	COSMIC,^27^ FOETAL,^17^ RECIPAL^15^ ZAPPS^26^	–	–
Past medical and obstetric history	10 (71.4%)	COSMIC,^27^ MiPADD,^20^ ENID,^18^ FOETAL,^17^ RECIPAL,^15^ AMANHI,^10^ NuPED,^16^ ZAPPS,^26^ HeLTI,^22^ PRECISE^9^	2 (20.0%)	COSMIC,^27^ ZAPPS^26^	–	–
Clinical history and examination	7 (50.0%)	IPTp +,^21^ ENID,^18^ COSMIC,^27^ FOETAL,^17^ RECIPAL,^15^ ZAPPS,^26^ PRECISE^9^	–		–	–
Pregnancy dating by ultrasound	8 (57.1%)	ENID,^18^ FOETAL,^17^ RECIPAL,^15^ AMANHI,^10^ INTERBIO-21st^19^ NuPED,^16^ ZAPPS,^26^ PRECISE^9^	3 (37.5%)	FOETAL,^17^ RECIPAL,^15^ ZAPPS^26^	–	–
Maternal outcomes						
Maternal mortality	13 (92.9%)	IPTp +,^21^ STOPPAM,^24^ COSMIC,^27^ MiPADD,^20^ ENID,^18^ FOETAL,^17^ RECIPAL,^15^ AMANHI,^10^ INTERBIO-21st,^19^ NuPED,^16^ ZAPPS,^26^ HeLTI,^22^ PRECISE^9^	10 (76.9%)	IPTp +,^21^ COSMIC,^27^ MiPADD,^20^ ENID,^18^ FOETAL,^17^ RECIPAL,^15^ NuPED^16^	150 (80, 280) per 100,000 births	14.6%
Pregnancy hypertension	8 (57.1%)	STOPPAM,^24^ FOETAL,^17^ RECIPAL,^15^ AMANHI,^10^ INTERBIO-21st,^19^ NuPED,^16^ ZAPPS,^26^ HeLTI,^22^ PRECISE^9^	2 (25.0%)	RECIPAL,^15^ ZAPPS^26^	3.4% (2.6%, 4.4%)	NA
Anaemia	13 (92.9%)	IPTp +,^21^ STOPPAM,^24^ COSMIC,^27^ MiPADD,^20^ ENID,^18^ FOETAL,^17^ RECIPAL,^15^ AMANHI,^10^ INTERBIO-21st,^19^ NuPED,^16^ ZAPPS,^26^ HeLTI,^22^ PRECISE^9^	8 (61.5%)	IPTp +,^21^ COSMIC,^27^ MiPADD,^20^ ENID,^18^ FOETAL,^17^ RECIPAL,^15^ NuPED,^16^ ZAPPS^26^	37.4% (22.0%, 55.9%)	99.7%
Malaria	10 (71.4%)	IPTp +,^21^ STOPPAM,^24^ COSMIC,^27^ MiPADD,^20^ FOETAL,^17^ RECIPAL,^15^ AMANHI,^10^ INTERBIO-21st,^19^ ZAPPS,^26^ PRECISE^9^	6 (60.0%)	IPTp +,^21^ COSMIC,^27^ MiPADD,^20^ FOETAL,^17^ RECIPAL,^15^ ZAPPS^26^	5.2% (0.9%, 24.4%)	99.5%
Long term outcomes	1 (7.1%)	HeLTI^22^	0 (0.0%)	–	–	–
Perinatal and infant outcomes						–
Preterm birth	12 (85.7%)	IPTp +,^21^ STOPPAM,^24^ COSMIC,^27^ MiPADD,^20^ FOETAL,^17^ RECIPAL,^15^ AMANHI,^10^ INTERBIO-21st,^19^ NuPED,^16^ ZAPPS,^26^ HeLTI,^22^ PRECISE^9^	6 (42.9%)	IPTp +,^21^ COSMIC,^27^ MiPADD,^20^ FOETAL,^17^ RECIPAL,^15^ NuPED^16^	4.3% (2.5%, 7.5%)	95.7%
Foetal growth restriction	9 (64.2%)	ENID,^18^ FOETAL,^17^ RECIPAL,^15^ AMANHI,^10^ INTERBIO-21st,^19^ NuPED,^16^ ZAPPS,^26^ HeLTI,^22^ PRECISE^9^	3 (33.3%)	ENID,^18^ FOETAL,^17^ RECIPAL^15^	14.6% (8.4%, 24.1%)	94.2%
Stillbirth	13 (92.9%)	IPTp +,^21^ STOPPAM,^24^ COSMIC,^27^ MiPADD,^20^ ENID,^18^ FOETAL,^17^ RECIPAL,^15^ AMANHI,^10^ INTERBIO-21st,^19^ NuPED,^16^ ZAPPS,^26^ HeLTI,^22^ PRECISE^9^	7 (53.8%)	IPTp +,^21^ STOPPAM,^24^ COSMIC,^27^ MiPADD,^20^ ENID,^18^ FOETAL,^17^ RECIPAL^15^	20 (15, 28) per 1000 births	84.6%
Neonatal death	12 (85.7%)	IPTp +,^21^ Seychelles Child Development Study,^23^ COSMIC,^27^ MiPADD,^20^ FOETAL,^17^ RECIPAL,^15^ AMANHI,^10^ INTERBIO-21st,^19^ NuPED,^16^ ZAPPS,^26^ HeLTI,^22^ PRECISE^9^	5 (41.7%)	IPTp +,^21^ Seychelles Child Development Study,^23^ COSMIC,^27^ MiPADD,^20^ FOETAL^17^	11 (6, 20) per 1000 livebirths	89.1%
Long term outcomes	3 (21.4%)	Seychelles Child Development Study,^23^ ENID,^18^ HeLTI^22^	1 (33.3%)	Seychelles Child Development Study^23^	–	–

*Inclusive of studies planning to report on these variables/outcomes, but for which data collection is still ongoing.

†Denominator based on number of studies collecting corresponding outcome.

‡Based on random effects logistic regression model.

## Discussion

### Summary of findings

All fields of clinical research increasingly acknowledge the importance of pathways that can only be studied with biological analyses, including modern ‘omics techniques, such as genomics, transcriptomics and metabolomics—with epigenomics becoming increasingly popular. Maternal health and pregnancy studies are no exception, with many studies beginning to assess the significance of these relationships.^5–8^ Until recently, the collection of such samples and in-depth biological investigation have taken place almost exclusively in high-income settings and populations.

This systematic review set out to determine to what extent biobanking has been implemented in pregnancy studies in SSA. In many lower resourced settings in SSA, women face distinct challenges and a higher burden of morbidity and mortality than those in higher resourced settings.^11 12^ The findings of this review suggest that biological sampling is gaining popularity in SSA, as all the included cohorts either completed data collection within the past decade, or have data collection ongoing. The included studies can be broadly grouped into two categories: clinical studies or trials that include biological sampling, or those with a primary focus on biobanking and discovery science. For example, both the Community-based Scheduled Screening and Treatment of Malaria in Pregnancy for Improved Maternal and Infant Health Consortium (COSMIC), and Strategies TO Prevent Pregnancy-Associated Malaria (STOPPAM) studies reported malaria as the primary outcome, but undertook biological sampling for malaria testing, prevalence and treatment effects.^19 22^ Many similar malaria studies were excluded as blood samples were not banked for future research (or not indicated for this purpose). This was also true of many HIV and anaemia-focused cohorts, where sampling was for point-of-care testing or immediate biomarker assessment only.

This review has also revealed that for the most part, SSA-based biobanking pregnancy studies do not operate in isolation and are often part of larger global consortia (INTERBIO-21st, Alliance for Maternal and Newborn Health Improvement (AMANHI), Zambian Preterm Birth Prevention Study/Global Alliance to Prevent Prematurity and Stillbirth (ZAPPS/GAPPS), Healthy Life Trajectories Initiative (HeLTI), Pregnancy Care Integrating Translational Science, Everywhere (PRECISE)^9 10 17 21 27^). INTERBIO-21st and AMANHI, for example, are comprehensive in scope, collecting data on a wide variety of clinical outcomes and addressing possible associations with biomarkers.^10 27^ These cohorts both contribute to larger global consortia—including partnerships with South Asia—as well as Brazil and the UK, in the case of INTERBIO-21st.^27^ To date, ‘at scale’ biomarker or ‘omics data are unavailable for either of these cohorts, but forthcoming ‘between-country’ comparisons of disease pathways are likely to offer the optimal understanding of regional variability in disease aetiology. Further, the above-mentioned biobanks^9 10 17 21 27^ are harmonised in terms of informed consent (using a tiered informed consent model), and collection, processing and storage SOPs, which will enable future pooling of samples (currently being funded through the Missed Opportunities in Maternal and Infant Health consortium).^9^ This harmonisation across operations can provide more ample opportunity for future collaboration, increases the likelihood that banked samples will be used—as well as increases the comparability of findings across studies.

The most commonly collected samples in the included studies were maternal venous blood, cord blood and urine. These are all simple to collect and have high analytical utility. Blood was collected in 92.0% of women from the five studies reporting. It is encouraging that placenta samples were taken in 11 of the 14 studies as these are a valuable resource for future analyses. In the studies with publicly available data, both cord blood and placenta samples were ascertained in ~70% of subjects. This represents a further success, given the challenges associated with following women to delivery, collecting at time-of-delivery samples and transporting samples to the laboratory in time for adequate processing and biobanking.^28^

Most cohorts captured standard maternal and perinatal outcomes. Preterm birth (based on estimated gestation at birth) was a common primary or secondary outcome, having a high incidence and reported associations with several biomarkers (eg, alpha-fetoprotein and C-reactive protein).^29^ Maternal mortality was another, with the pooled maternal mortality ratio from six studies being 150 per 100 000 (95% CI 80 to 280), however lower than previously reported rates for SSA as a whole.^30^ Similarly, pooled stillbirth and neonatal death rates were lower than those reported for the region. This may indicate selection bias in the participant populations, lack of follow-up to 42 days post partum or perhaps a consequence of engagement in research, where more comprehensive obstetric care may be available.

### Biobanking in context

The unique challenges faced by health systems in SSA highlight the potential for biobanking studies on the continent to address gaps in public and global health domains. Further, African-based and African-led biorepositories offer opportunities for training, capacity building and contributions to health and scientific infrastructure. However, the establishment of biobanking studies is not without its own set of challenges. This can involve complex processes for governance and culturally appropriate data and sample collection. The movement (particularly across borders) and storage of data and samples can be both ethically and logistically complex. Resource and supply-heavy operating procedures, as well as funding longevity, pose additional challenges.^31 32^ Robust partnerships and collaborations between proposed and existing biobanking initiatives may offer innovative strategies for expanding research and research capacities, while minimising the above-mentioned challenges. However, inadequate biobanking regulatory frameworks and insufficient global governance structures continue to be issues of debate.^31 32^ Furthermore, in some countries anxiety around use of samples for future research has led to regulatory barriers with, in some cases, limits being imposed on the number of years samples can be stored.^33^ As such, it is essential that future biobanking initiatives and partnerships be aware of these potential pitfalls, and equitably benefit all parties involved.

The new focus on investigation in SSA and other lower and middle-income countries clearly parallels the burden of disease. While pregnancy cohorts for discovery science have previously been concentrated on populations in the global North, the shift to the global South is appropriate due to the recognition that differing biological ‘routes’ may lead to the same adverse outcome, depending on geographical setting.^34^ Examples include the Prematurity Reduction by Pre-eclampsia Care study in Brazil which will use biomarkers to assess risk factors for pre-eclampsia in women across six Brazilian cities.^34^ Similarly, in India—and using an integrated approach (clinical, epigenetics, epigenomics, genomics, proteomics and microbial)—the Interdisciplinary Group for Advanced Research on Birth Outcomes-DBT India Initiative has followed >4000 women throughout their pregnancies to determine potential predictors for preterm birth.^30^ The PRECISE Network in West (The Gambia), East (Kenya) and Southern (Mozambique) SSA^9 35 36^ was established to understand the social, clinical and biological determinants of healthy and complicated pregnancy outcomes. These studies highlight the potential for pregnancy cohorts and high throughput technologies to improve the understanding of common pregnancy complications in the locations where they occur, offering the greatest opportunity for appropriately targeted interventions.

Finally, given the increased emphasis and shifting focus of global health research to the novel COVID-19 pandemic, biobanks have the potential to be an important and unique resource for furthering our understanding of the risks and implications of this disease in pregnancy.

### Strengths

To our knowledge, this is the first systematic review to assess the scope of biobanking studies in prospective pregnancy cohorts in SSA, and to report on the diversity of samples collected. While a previous review published in 2011 included both retrospective and prospective studies, this review reported only on blood sampling, and reported high attrition rates in cohorts, while also highlighting the absence of large-scale cohorts.^37^ Our review, comprehensive in inclusion of scientific databases, sampling protocols and secondary sources, illustrates a rapidly evolving landscape over the intervening years.

### Limitations

The most significant challenge encountered was the infrequent use of the term ‘biobanking’. While collection and storage of biological samples for future purposes is common, few studies explicitly referred to this process as ‘biobanking’. Extension of our definition of biobanking to include studies mentioning collection and storing of samples for future use, ancillary purposes or substudies may have led to exclusion of those with no explicit mention of sample storage. The database search also presented challenges, as the MeSH terms and keywords often did not accurately reflect the specific study information. Based on discussion with members of the global health community, there may be additional studies or internal quality improvement projects taking place in SSA that have not yet reported their biobanks. Also, some studies ineligible for inclusion are newly established, and consequently, their protocols are not yet published. Therefore, the total number included may have been underestimated. Notwithstanding this, our comprehensive search was unlikely to have missed well-established cohorts.

### Recommendations for future research

The cohorts identified provide a full picture of the state of biobanking in maternal health research in SSA and offer clear indications to where gaps exist, and where they are being addressed. In particular, we found very few studies with detailed long-term follow-up of mother–infant pairs or studies which include comprehensive neonatal samples. Only one study included pre-pregnancy data, and with an increasing global focus on preconception health this focus is likely to become more common.^33^ Given that few studies have been, or are, collecting hair, stool, vaginal swabs, tissue and breast milk samples, the biological data and research findings that can be obtained from these sample types should not be considered a representative overview for SSA. Depending on feasibility and the objectives of future studies, collection of these sample types may be warranted for inclusion. Finally, as the majority of SSA countries are not included in these studies, and given the relatively lower maternal and perinatal mortality rates compared with regional estimates,^11 12^ it is possible that populations of SSA women who are at higher risk are under-represented. Also, the majority of these studies include women who deliver in health facilities, and consequently miss many women who are unable to deliver in these facilities (eg, those women from rural or remote locations or without access to transport). Future studies which include these most vulnerable populations of women constitute a priority.

Further, publications by existing cohort studies will be useful in identifying those available for future use. Publicly available protocols accessible early in a study’s trajectory can offer valuable information to the research and global health community and are most useful when published at the time of research activities, not with final results. Standardising definitions of variables and outcomes, and establishing biorepository standards will catalyse the acceleration of discovery science—by facilitating the creation of virtual cohorts that bring together otherwise disparate studies. Given the time and effort required from both researchers and study participants to obtain, store and analyse biological data and samples, it is crucial that they are used equitably, ethically and to their full capacity, even once primary objectives of studies have been assessed and completed. To facilitate collaborative research, we recommend that SSA pregnancy cohorts are registered on one of the international birth cohort registries, such as birthcohorts.net, or consider developing an SSA-specific registry.

## Conclusion

An increasing number of pregnancy studies in SSA include biorepositories, with maternal, cord blood, and placenta samples being those most commonly collected and stored. Future research will be most beneficial and impactful if it is complementary to these existing biobanks. In addition, studies focusing on maternal–infant dyads with a view to understanding the life course of health and disease, and in under-represented geographic regions, have the potential to make meaningful contributions to understanding the early life origins of disease. These activities, taken together, should lend strength to global health partnerships and alliances.
