# Supplementary material for: Pregnancy cohorts and biobanking in sub-Saharan Africa: a systematic review
Source: BMJ Glob Health. 2020 Nov 26;5(11):e003716. doi: 10.1136/bmjgh-2020-003716 (PMC7692823; doi:10.1136/bmjgh-2020-003716)
Supplement: Supplementary data [file bmjgh-2020-003716supp001.pdf]

Supplementary Tables

Table S1: PRECISE Network

Umberto D'Alessandro, Anna Roca, Hawanatu Jah, Ofordile Oguchukwu, Andrew Prentice, Melisa Martinez-Alvarez, Brahima Diallo, Adbul Sesey, Kodou Lette, Alpha Bah, Chilel Sanyang, Marleen Temmerman, Angela Koech Etyang, Peris Musitia, Mary Amondi, David Chege, Patricia Okiro, Geoffrey Omuse, Sikolia Wanyonyi, Esperança Sevene, Paulo Chin, Corssino Tchavana, Salésio Macuácua, Anifa Vála, Helena Boene, Lazaro Quimice, Sonia Maculuve, Eusebio Macete, Inacio Mandomando, Carla Carillho, Peter von Dadelszen, Laura A. Magee, Meriel Flint-O'Kane, Rachel Craik, Amber Strang, Marina Daniele, Donna Russell, Tatenda Makanga, Liberty Makacha, Yolisa Dube, Newton Nyapwere, Lucilla Poston, Jane Sandall, Rachel Tribe, Andrew Shennan, Sophie Moore, Tatiana Salisbury, Ben Barratt, Lucy Chappell, Sean Beevers, Kate Bramham, Aris Papageorgiou, Alison Noble, Hannah Blencowe, Veronique Filippi, Joy Lawn, Matt Silver, Matthew Chico, Judith Cartwright, Guy Whitley, Sanjeev Krishna, Marianne Vidler, Jing (Larry) Li, Jeffrey Bone, Mai-Lei (Maggie) Woo Kinshella, Beth A. Payne, Domena Tu, Warancha Tumtaweetikul, William Stones, Marie-Laure Volvert

Table S2. Finalised keyword and MeSH search terms for database searches.

**Biobanking**	
	(biological banks/ or biospecimen banks/ or biological specimen banks/ or tissue banks/ or vaginal swab/ or blood banks/ or tissue sample/ or blood sample/ or vaginal smear/ or breast milk bank/ or lactation sample/ or biopsy/ or blood specimen collection/ or tissues/ or body fluid compartments/ or membranes/ or Biomarkers/ or milk bank/ or biorepository/ or biological samples/) not animals
	(genomics/ or epigenomics/ or developmental biology/ or embryology/ or genetics/ or genetic research/ or genetics, microbial/ or human genetics/ or genetics, medical/ or Genome, Bacterial/ or phenotype/) not animals
	biological specimen banks or blood banks or milk banks or tissue banks or biopsy or vaginal smears or blood specimen collection or Biomarkers or lactation sample or biological samples or epigenomics or developmental biology or embryology or genetics or genetic research or microbial genetics or human genetics or medical genetics or genome or phenotype or lactation sample)
**Maternal health**	
	reproductive health/ or women's health/ or maternal health/
	reproductive health or women's health or maternal health or perinatal or antenatal
	nutrition, pregnan* or prenatal nutrition
	fetal mortality/ or infant mortality/ or perinatal mortality/ or maternal mortality/ or mortality, premature/ or pregnancy rate/ or birth rate/
	fetal mortality or infant mortality or perinatal mortality or maternal mortality or mortality, premature or pregnancy rate or birth rate
	hypertension, pregnancy-induced/ or pre-eclampsia/ or preeclampsia/ or eclampsia/ or placental diseases/ or hellp syndrome/ or haemmorhage, post-partum/ or hemmorage, post-partum/ or haemmorhage, postpartum/ or hemmorage, postpartum/ or still birth/ or still-birth/ or pre-term delivery/ or preterm delivery/ or sepsis/ or maternal death/ or pregnancy complications/ or preterm birth/ or fetal growth retardation.mp
	(biological specimen banks or blood banks or milk banks or tissue banks or biopsy or vaginal smears or blood specimen collection or Biomarkers or lactation sample or biological samples or epigenomics or developmental biology or embryology or genetics or genetic research or microbial genetics or human genetics or medical genetics or genome or phenotype or lactation sample)
**Geography**	
	Sub-saharan Africa/ or Sub-Sahara Africa/ or Africa South of the Sahara/
	(sub-sahar* Africa or south* Africa or west* Africa or east* Africa).mp.
	(Angola or Benin or Botswana or Burkina Faso or Burkina Fasso or Burundi or Cameroon or Cameroons or Cameron or Camerons or Cape Verde or Cabo Verde or Central African Republic or Chad or Comoros or Comoro Islands or Comores or Mayotte or Congo or Zaire or Cote d'Ivoire or Ivory Coast or Djibouti or French Somaliland or Eswatini or Eritrea or Ethiopia or Gabon or Gabonese Republic or Gambia or Ghana or Guinea or Guinea-Bissau or Kenya or Lesotho or Basutoland or Liberia or Madagascar or Malawi or Nyasaland or Mali Mauritania or Mauritius or Mozambique or Namibia or Niger or Nigeria or Rwanda or Ruanda or Sao Tome or (Sao Tome and Principe) or Senegal or Seychelles or Sierra Leone or Somalia or South Africa or Sudan or South Sudan or Swaziland or Tanzania or Togo or Uganda or Zambia or Zimbabwe or Rhodesia).mp.

Table S3: Newcastle-Ottawa quality assessment scale (NOS) for each included study. A * indicates 'yes', a blank cell 'no' and NA indicates not applicable based on existing cohort publications.

Study	Selection (/4)				Comparability (/2)		Outcome (/3)			Total (/9)
	Representativeness	Selection	Ascertainment of exposure	Outcome not present at start of study	Control study primary	Control study secondary	Assessment	Adequate follow up time	Follow up data	
IPTp +(21)	*	*	*	*	*	*	*	*	*	9
STOPPAM (24)	*	*	*	*	*		*	*	*	8
Seychelles Child Development Study (23)	*	*	*	*	*	*		*	*	8
MiPADD (20)	*	*	*	*	*	*	*	*	*	9
ENID (18)	*	*	*	*	*	*	*	*	*	9
COSMIC (27)	*	*	*	*	*	*	*	*		8
FOETAL (17)	*	*	*	*	NA	NA	*	*	*	7
RECIPAL(15)	*	*	*	*	*	*	*	*	NA	8
AMANHI (10)	*	*	*	*	NA	NA	*	*	NA	6
INTERBIO-21st (19)	*	*	*	*	NA	NA	*	*	NA	6
NuPED (16)		*	*	*	*	*	*	*		7
ZAPPS (26)	*	*	*	*	NA	NA	*	*	NA	6
PRECISE (9)	*	*	*	*	NA	NA	*	*	NA	6
HeLTI (22)	*	*	*	*	NA	NA	*	*	NA	6
